# English as It Is Read in the Grady Hospital

**Published:** 1893-03

**Authors:** 


### ENGLISH, AS SHE IS READ, IN THE GRADY HOSPITAL.

The medical profession takes no stock in the ordinary family quarrels at the Grady Hospital. But when an issue comes before the public, involving the good name and ethical status of the professional staff of that institution, it comes home to us as journalists.

It is announced semi-officially that the following resolution was originated by the Board of Surgeons, with the request that it be adopted:

"Resolved, That no officer or employee of the Grady Hospital shall give any information to the newspapers regarding the condition of patients or the the treatment of the same, or of any surgical operation performed by any surgeon in attendance at the hospital."

That the surgical staff should call for the passage of the above resolution, seems to outsiders very strange. But the remarkable phase of this proceeding is that when the superintendent undertakes to comply with it, he is told by one of the Board of Trustees that it does not mean what it plainly states. It appears from his explanation that it must have been enacted with mental reservation or is intended to apply to a portion of the officers of the hospital and not to others.

The intimation, that it was deemed the proper thing on account of some jealousies that have arisen among surgeons over newspaper notices of operations performed by members of the staff, does not, of course, apply to surgeons not connected with the institution. It is generally understood that it is a wholesale reflection upon the officers of the Grady Hospital, and that it would be appropriately resented by the withdrawal of every member of the visiting staff from the service.

It is not a matter of surprise that difficulties should be encountered in the administration of the hospital, when such regulations and explanations as are brought to the notice of the public at present, are in full operation.

It strikes us that a good off-set to the action of the Board of Trustees of the Grady Hospital in this instance would be for the Council and Board of Aldermen of the City of Atlanta to prohibit, under penalties, the entire medical profession from reporting or causing to be reported, the cases under their treatment or who have been operated upon by them. It would be in keeping with this high ethical standard that the names of prominent citizens, who come to our city, with a view to consult specialists, should cease to appear in the columns of our newspapers. J. McF. G.

---

RESOLUTIONS OF RESPECT PASSED ON THE DEATH OF DR. W. S. HART, A GRADUATE OF THE ATLANTA MEDICAL COLLEGE.—The death of Dr. W. S. Hart, one of the most prominent graduates of the Atlanta Medical College, which occurred last Friday afternoon in this city, has been the cause of very general sorrow among his many friends in Atlanta.

His death occurring at this particular time, near the close of his collegiate term, is rendered all the more pathetic, and throws a veil of sorrow over the festivities of commencement.

The following set of resolutions was drawn up by a committee of his classmates last Friday afternoon :

"Whereas, It has pleased Almighty God to take from our midst our friend and former collegemate, Dr. Smith W. Hart, be it

"Resolved, That we, the senior class of the Atlanta Medical College, tender our deepest sympathies to his grief stricken parents in this their hour of affliction.

Resolved further, That we have these resolutions published in the Atlanta Constitution and SOUTHERN MEDICAL RECORD. We also request that a copy of the same be sent to his parents.

W. H. BRYAN,
H. S. WRIGHT,
C. J. VAUGHAN,
Committee."

---

Having used MCARTHUR'S CHEMICALLY PURE Syrup of the Hypophosphites of Lime and Soda for some time in my practice, it affords me pleasure to recommend it to my patients who are suffering from incipient phthisis, chronic bronchitis and other pulmonary affections. In all wasting diseases I think it a most reliable remedy. It increases appetite and promotes digestion.—*David F. Drew, M. D., Councillor Massachusetts Medical Society.*

